# Conductometric Sensor for PAH Detection with Molecularly Imprinted Polymer as Recognition Layer †

**DOI:** 10.3390/s18030767

**Published:** 2018-03-03

**Authors:** Usman Latif, Liu Ping, Franz L. Dickert

**Affiliations:** 1 Department of Analytical Chemistry, University of Vienna, Währinger Strasse 38, Vienna A-1090, Austria; liupingsjj@sjtu.edu.cn; 2 Interdisciplinary Research Centre in Biomedical Materials (IRCBM), COMSATS Institute of Information Technology (CIIT), Lahore 54000, Pakistan; usmanlatif@ciitlahore.edu.pk

**Keywords:** conductometric sensor, screen-printing, interdigital gold electrodes, molecularly imprinted polymers, polycyclic aromatic hydrocarbons

## Abstract

A conductometric sensor based on screen-printed interdigital gold electrodes on glass substrate coated with molecularly imprinted polyurethane layers was fabricated to detect polycyclic aromatic hydrocarbons (PAHs) in water. The results prove that screen-printed interdigital electrodes are very suitable transducers to fabricate low-cost sensor systems for measuring change in resistance of PAH-imprinted layers while exposing to different PAHs. The sensor showed good selectivity to its templated molecules and high sensitivity with a detection limit of 1.3 nmol/L e.g., for anthracene in water which is lower than WHO’s permissible limit.

## 1. Introduction

There is a huge demand for environmental monitoring technologies due to increasing environmental pollutants and their adverse health effects. PAHs are ubiquitous environmental contaminants mainly from incomplete combustion of various materials, especially from petroleum. PAHs are a group of persistent organic pollutants and bioaccumulatives [1]. They are moderate-to-highly toxic due to their mutagenic, teratogenic, and carcinogenic properties [2,3]. PAHs contains at least two aromatic rings. Anthracene, a PAH consisting of three fused benzene rings, is deleterious to human health and can cause lung, liver, lymph, and skin disorders [4]. The prime importance has been given, in the latest European Water Framework Directives (Directives 2000/60/EC, 2006/118/EC, and 2006/11/EC), to develop those analytical tools which are useful for sensitive and on-site detection of pollutants in environmental waters [5]. 

Existing techniques for detecting PAHs rely on bulky instruments which require sample preparation, its isolation and large volume of organic solvents that make these methods very time consuming and costly. There are some reports about coupling selective extractions with instrumental analysis to detect PAHs [6,7,8,9,10,11,12,13]. Trace analytical methods for complex matrices rely on efficient sample enrichment and selective assays. These methods are very sensitive but ill-suited for on-site monitoring of pollutants due to their limitations. Chemical sensors have emerged as a powerful tool for special analytes using artificial recognition sites [14]. In the search for new recognition techniques, molecular imprinted polymers (MIP) have opened a way to a novel type of selective sorbent with attractive properties. On-line monitoring of small organic pollutants in waters is feasible with MIPs [15,16,17,18,19,20,21]. Membrane-based conductometric sensors for various target compounds have also been reported [22,23,24]. Imprinted layers of modified non-porous or porous particles, films, or other materials can be used to detect various analytes. Non-covalent imprinting can be easily performed for small molecules with no functionality such as PAHs. The imprinting effect depends preferably on van der Waals forces between template and self-organizing polymers.

In this study, MIP based conductometric sensor has been developed for anthracene with a detection limit of 1.3 nmol/L which is lower than WHO’s permissible limit of PAHs (3.93 nmol/L) [25]. 

## 2. Materials and Methods

Pre-cleaned, plain, cut edges microscope glass slides (76 mm × 26 mm × 1 mm) were purchased from Marienfeld. Gold paste (GGP 2093, 12%) for electrode printing was received from Heraeus, Germany. The isolating paste (Urethan 71) was purchased from CRC Industries. PAHs were purchased from Fluka and Sigma. Bisphenol A (BPA), 4,4′-methylenediphenyldiisocyanate (MDI), tetrahydrofuran (THF) and dimethylsulfoxide (DMSO), and phloroglucinol (PG) were purchased from Merck and Fluka. 3-aminopropyltriethoxysilane was obtained from ABCR. Analytical grade chemicals were obtained and used as received.

### 2.1. Fabrication and Surface Modification of Electrodes

**Figure 1:**
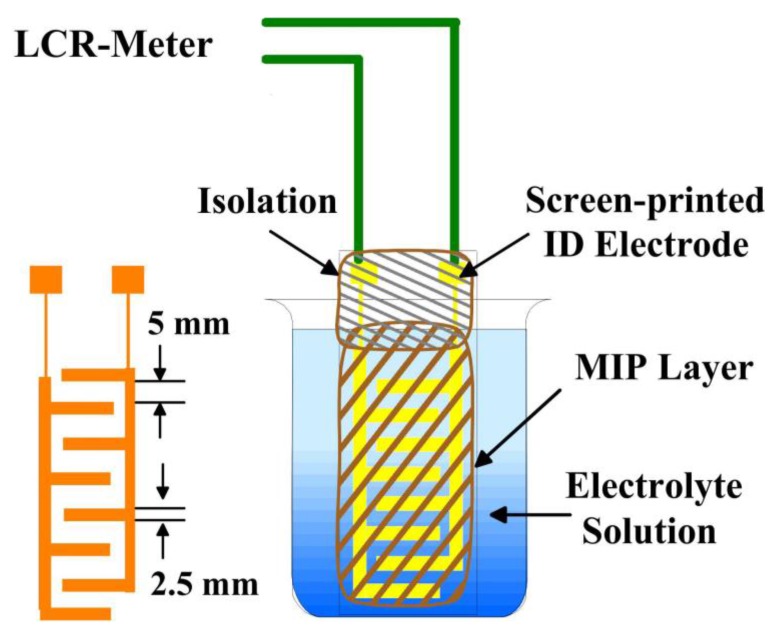
IDT was fabricated by screen printing IDE of gold on glass substrate. IDT was coated with sensitive layer and LCR meter was used to measure resistance of sensitive layer by placing it in glass cell filled with electrolyte solution.

Interdigital electrodes (IDE) were screen-printed on a glass substrate (microscope slide) to fabricate an interdigital transducer (IDT). The equivalent circuit diagram and working principle of IDT was already explained in our previous paper [24]. Screen-printed interdigitated gold electrode-based sensor systems have been used for variety of analytes [26,27,28]. The desired structure of interdigital electrodes was fabricated on the substrate via screen printing with commercial gold paste. The type of the screen was 120-34W PW of Sefar Inc. (Thal, Switzerland). The electrode structure was designed by photo lithography. All organic components present in gold paste were removed by heating at 500 °C for 4 h. The advantage of this procedure is based on the robustness of the gold layers. The pattern and dimensions of the IDEs structure on transducer surface is shown in Figure 1. 

The prepared transducer was cooled down to room temperature, then washed in an ultrasonic water bath for 5 min and dried. The IDT was placed in a boiling mixture of chloroform and ethanol (1:1 v/v) for at least 10 min for washing. Afterwards, IDT was treated with hot lithium hydroxide solution (0.2 mM) for 10 min, rinsed with distilled water and dried. Then, IDT was dipped in a solution of 3-aminopropyl-triethoxysilane in ethanol for 1–2 min followed by rinsing with ethanol. The silanized structure was cured for 10 min at 110 °C. 

Molecular imprinted polyurethane layers were synthesized by following the procedure from our previous paper [14]. BPA, PG, and MDI containing 30% of the respective triisocyanate (mixture of isomers) and 5–10% of PAH as imprint were dissolved in THF to prepare PAH-imprinted polyurethane layer. The pre-polymerized solution (70 °C for 1 h) from above mentioned mixture was spin-coated on IDT surface. A mixture of monomers, cross-linker, and template was heated at 70 °C until its gelation point which is called as pre-polymer. This pre-polymer was applied to transducer surface to form a rigid layer after complete polymerization. The height of the coating can be controlled by the rotation speed of the spin coating apparatus, which was home-made. Additionally, the viscosity of the pre-polymer can be varied by dilution, which has the same effect as higher rotation speed. The highly cross-linked prepolymer formed a rigid clear transparent homogenous layer on IDT surface after complete condensation. The surface condition and heights of layers were analyzed by using atomic force microscope (VEECO Nanoscope IVa) in contact mode. The electrodes were connected with Hewlett-Packard 4284 A precision LCR meter, the uncoated area was finally isolated with urethane 71. The reference transducer was prepared with non-imprinted polyurethane in exactly same way (these are called ‘non-imp. electrodes’). 

### 2.2. Removal of the Template Molecule

We have used different ways to remove imprinted molecule from MIP layers such as washing with toluene, rinsing with water, and evaporating using hot air. The imprinted layer, containing high boiling point PAHs or low evaporation rates, was washed by toluene and water. It should be very careful to control the washing time of toluene, otherwise toluene can damage the cavity structure in MIPs layer. Whereas, rest of PAHs (templates) were easily removed by evaporating at elevated temperatures. The template molecules were evaporated from MIPs by using hot air (28 °C). It should be given enough evaporating time to generate diffusion pathways while removing template molecules, during polymerization, otherwise diffusion pathways will close (if template evaporate immediately) when polymer is still rearranging itself. The imprinted polymer layer containing 10% naphthalene as template, with different evaporating times was examined by luminescence spectrometer (Perkin-Elmer LS-50B). The results can be seen in Table 1. This method is suitable for removing both naphthalene and anthracene from MIP layer. In general, cavities in MIPs were almost fully recovered after 4 h of evaporation. The imprinted layers were stored in an evacuated desiccator to accelerate evaporation

### 2.3. Measurements 

The resistance of the sensitive layer (after template removal) coated on IDT was measured by placing it in a glass cell having a supporting electrolyte as shown in Figure 1. The stock solution of analytes was added into the glass cell having 50 mL of supporting electrolyte to obtain final medium. Hewlett-Packard 4284A precision LCR meter (20 Hz–1 MHz) was operated in an AC mode to measure resistance of sensitive layers. The surface and the thickness of the MIP layers were measured with a VEECO Nanoscope IVa atomic force microscope.

## 3. Results and Discussion

### 3.1. Thickness of MIP Layers

The polyurethane layers were imprinted with anthracene and applied to transducer surface in a height ranging from 250 nm to approximately 1 μm. The transducer surface was covered with MIP layers by spin-coating prepolymer solution. Required thickness was achieved by adjusting speed according to solution viscosity. All spin-coated layers were homogeneous, transparent, and highly stable. The absolute resistance due to analyte enrichment increases with layer thickness. A layer in a height of 250 μm will have a resistance of approximately 20 kΩ, whereas coatings in a thickness of some micro meter show resistances of several MΩ. The stated enrichment process with blocked diffusion channels is a plausible reason for this sensor effects [29]. In summary, the thickness of sensitive layer is an essential factor in conductometric measurements. Here, 100 nm up to 1 μm layers are considered as an optimum thickness of PAH imprinted polyurethane layer in this conductometric sensor measurements, since these coatings were rigid without cracks.

**Figure 2:**
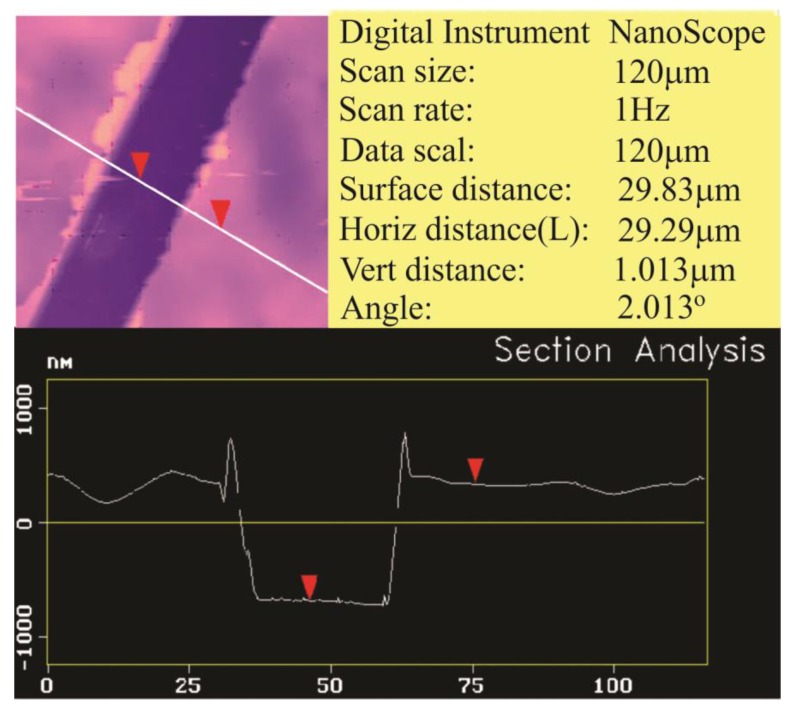
The thickness of 10% anthracene imprinted polymer layer measured by contact mode of AFM.

The surface morphology of sensor coatings was analyzed by using AFM in contact mode. Figure 2 shows a 10% anthracene imprinted homogeneous polyurethane layer. This depicts section analysis of scratched surface layer to measure its thickness, which is around 1 μm. 

### 3.2. Optimization of Measurement Conditions

**Figure 3:**
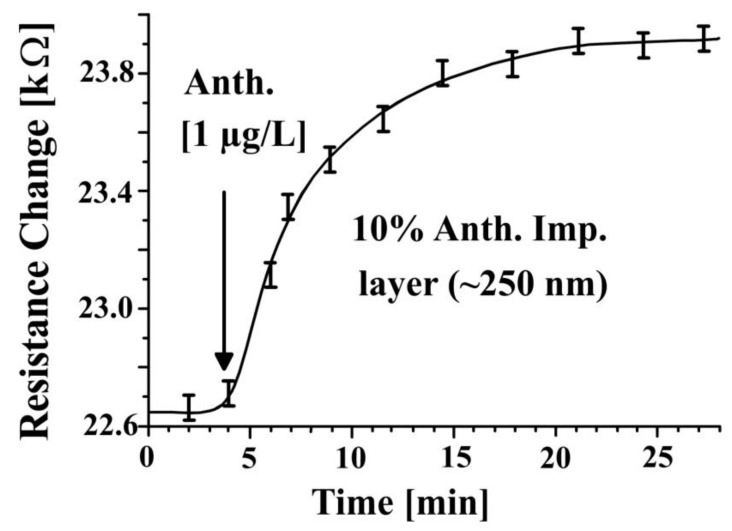
The resistance of 10% anthracene imprinted polyurethane layer (coated on IDT) measured with LCR meter at a frequency of 1 kHz while exposing to its template analyte solution. The resistance of layer increases with time due to blocking of diffusion channels.

The resistance of IDT covered with 10% anthracene imprinted polyurethane layer in aqueous NaCl solution (Figure 3) was measured with LCR meter at a frequency of 1 kHz. It was found that the resistance of MIP layer increased due to analyte incorporation in imprinted cavities of sensor coating as function of time. The increase in resistance is based on incorporation of analytes into the cavities of imprinted polymers. In this way, diffusion channels are closed and cavities are properly filled, which leads to an increase in resistance. The resistance of anthracene imprinted layer increases with time which means templated analyte (anthracene) is continuously diffused into a large number of cavities. In this experiment, 10% anthracene was used as template to synthesize imprinted polyurethane layer. The sensor is regenerated within approximately one hour. It can be seen from Figure 3 that signal intensity compared to error bar is approximately 13:1. The IUPAC designates the ratio 3:1 as significant response. Thus it can be concluded that a limiting concentration for the detection of anthracene is 5.6 nM (1 μg/L) 3/13 = 1.3 nM. 

**Figure 4:**
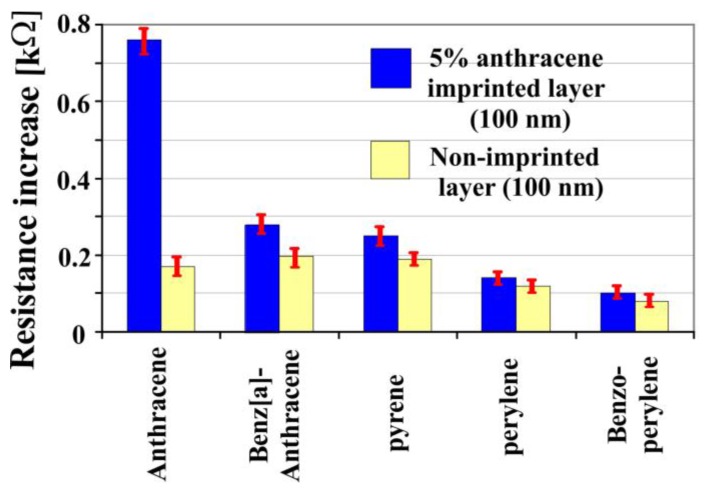
Anthracene imprinted layer was exposed to its templated analyte as well as other PAHs. The sensor showed higher sensor response to its templated analyte, but fewer responses were observed for other PAHs.

Selectivity of a 5% anthracene imprinted polyurethane layer (100 nm) based on the conductometric sensor is shown in Figure 4. The sensor layer yields the highest response to anthracene in comparison to other PAHs. Whereas minor sensor responses were observed for other PAHs, since they are not perfectly adapted or incorporated into receptor cavities of anthracene imprinted layer. Fewer changes occurred in resistance for the non-imprinted polyurethane layer (100 nm) due to absence of selective interaction sites.

### 3.3. Influence of Physical Parameters to Sensor Response 

The IDTs were coated with layers ranging from ~30 nm to 5 μm with different PAH imprinted polyurethanes. The results indicate that the layers with a height of 100 nm to 1 μm are best for resistance measurement in our experiment. The sensitivity of MIP layer to e.g., 2 μg/L analyte increased in parallel with the MIP layer thickness. Slightly enhanced response times, from 10–20 min, were observed while increasing the thickness of layer from 100 nm, 250 nm to 1 μm. The results were not reproducible while decreasing the thickness of layer below than 50 nm. Layers exceeding the height of 5 μm proved to be difficult in performing measurements due to high resistance.

The good wetting ability of polymer material is the basis of effects described. Thus, electrolytes can penetrate into the channels of the polymer materials. The ion mobility and its concentration will strongly influence the sensor basic response. 

### 3.4. Effect of Porogens

The use of porogens or porogenic solvents during polymerization is a best way to produce effective molecular recognition capabilities in polymers, especially in non-covalent molecular imprinting. The chemical and physical properties of porogenic additives during polymerization will influence the rigidity and functionality of MIPs. The porogens will induce porosity in the resulting microstructure, which will increase better accessibility of analytes to binding sites. The porogens should not compete with template during imprinting and should not form complexes with polymers. THF is different from PAH molecules and allows a good solubility of the polyurethane compounds.

**Figure 5:**
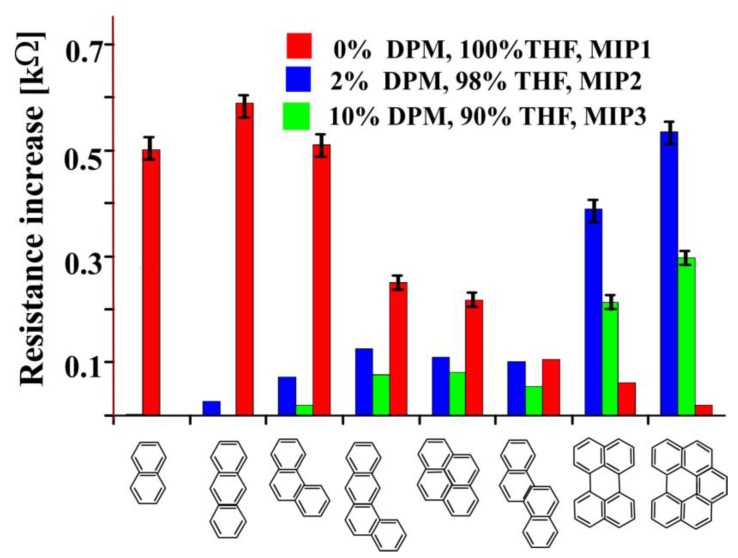
The effect of porogens on sensor responses of 5% anthracene imprinted polyurethane layer towards different PAHs.

For this purpose, diphenylmethane (DPM) was added to the mixture of pre-polymer. Three 5 *w*/*w*% anthracene imprinted polyurethane layers (MIP1, MIP2, MIP3) were prepared in a mixture of THF as solvent and DPM as porogen (the ratio of THF to DPM is 100:0, 98:2, and 90:10 respectively). These three anthracene imprinted layers show a characteristic response pattern to PAH molecules having different sizes and shapes as shown in Figure 5. Sensor coatings with MIP1 show appreciable responses to small PAHs such as naphthalene, anthracene, and phenanthrene. The molecular hollows will effectively bind the PAHs by which ion diffusion is hindered. In comparison to smaller PAHs, sensor response to bulky PAHs is lower. In accordance with these findings MIP2 layers show a pronounced sensitivity to bulky PAHs. The enlarged porosity will more strongly enrich bulky PAHs but not the small analogues. The further increase of porogens in MIP3 shows pronounced responses for bulky PAHs.

The resistances of sensitive layers were measured by placing them in a glass cell having supporting electrolyte. Thus, ion migration (from electrolyte) increases with porosity of polymer which results a decrease in resistance. The signal intensity, however, is smaller as for MIP2 since the increased porosity leads to a higher ion migration independent of the occupied or unoccupied cavities.

### 3.5. Stability and Reproducibility

The stability of MIP layer was tested over a period of two months by repeating measurements of its resistance to 2 μg/L anthracene in electrolyte solution. It was found that the sensor electrode which was stored at room temperature did not show any sensitivity loss over time.

The reproducibility of the sensor depends on the surface coating of electrode, the modification of screen-printed IDEs, and removal of template molecules. The reproducibility of MIP layer was achieved by controlling coating volume of fresh polymer on IDTs and speed of spin-coating. 

## 4. Conclusions

PAH imprinted polyurethane coated conductometric sensors based on screen-printed interdigitated electrode were developed. A screen-printed IDE on glass substrate (microscope slide) was proven to be a suitable low cost and easily fabricated conductometric transducer. A sensor coated with 1 μm 10% anthracene imprinted polymer layer shows the highest sensor response to the templated analyte. The detection limit to anthracene in 0.1 mM NaCl (as electrolyte) is 1.3 nM. The response time is about 20 min. The sensor response depends on the nature and concentration of the electrolyte used during resistive measurements. Thus, the starting resistance must be known. Furthermore, the temperature has to be kept constant during resistive measurement. Comparing with other sensors, it exhibits the advantages of easy fabrication, good storage stability, good regeneration ability, low cost, and easy miniaturization. 

## Figures and Tables

**Table 1:** Comparison of fluorescence intensity (Perkin-Elmer LS-50B—spectrometer) of 10% naphthalene imprinted polymer layer at different evaporation times.

Polymer	Non-Imprinted	10% Imprinted Layer				
Time [hour]	0	0	0.5	1.5	2.5	4
I/I_0_ [%]	2.5%	100%	50%	27%	21%	5%

I: fluorescence intensity of 10% naphthalene imprinted polymer layer after evaporation or non-imprinted layer; I_0_: fluorescence of freshly prepared 10% naphthalene imprinted polymer layer. 2.5%: percentage of fluorescence intensity of non-imprinted polymer layer to 10% PAH imprinted polymer layer (having same layer thickness).
